# Impact of Sex on Office White Coat Effect Tail: Investigating Two Italian Residential Cohorts

**DOI:** 10.1038/s41598-019-53109-1

**Published:** 2019-11-21

**Authors:** Xavier Humbert, Sophie Fedrizzi, Joachim Alexandre, Alessandro Menotti, Alain Manrique, Martino Laurenzi, Emmanuel Touzé, Paolo E. Puddu

**Affiliations:** 1 Normandie Université, UNICAEN, Département de médecine générale, 14000 Caen, France; 2 Normandie Université, UNICAEN, EA 4650, Signalisation, électrophysiologie et imagerie des lésions d’ischémie reperfusion myocardique, 14000 Caen, France; 3 Normandie Université, UNICAEN, CHU Caen Normandie, Service de pharmacologie, 14000 Caen, France; 4 Association for Cardiac Research, 00198 Rome, Italy; 5 Normandie Université, UNICAEN, CHU Caen Normandie, Service de médecine nucléaire, 14000 Caen, France; 6 Centro Studi Epidemiologici di Gubbio (CeSEG), 06024 Gubbio (Perugia), Italy; 7 Normandie Université, UNICAEN, Inserm U1237, CHU Caen Normandie, Service de Neurologie, 14000 Caen, France; 8 Sapienza University of Rome, Department of Cardiovascular, Respiratory, Nephrological, Anesthesiological and Geriatric Sciences, 00161 Rome, Italy

**Keywords:** Signs and symptoms, Cardiovascular biology

## Abstract

To assess the impact of sex on office white-coat effect tail (OWCET), the waning of systolic blood pressure (SBP) after its waxing during office visit, on the incidence of long-term major fatal and non-fatal events in two Italian residential cohorts [from the Gubbio Study and the Italian Rural Areas of the Seven Countries Study (IRA)]. There were 3565 persons (92 with missing data, 44% men, 54 ± 11 years) included in the Gubbio and 1712 men (49 ± 5 years) in the IRA studies. OWCET was defined as a decrease of ≥10 mmHg in SBP between successive measurements with slight measurement differences between the two cohorts. Cardiovascular (CVD), coronary heart disease (CHD) and stroke (STR) incidences were considered. Over an approximately 20-year follow-up, women with OWCET had an increased risk of CVD [HR: 1.591 (95%CI: 1.204–2.103)], CHD [HR: 1.614 (95%CI: 1.037–2.512)] and STR [HR: 1.696 (95%CI: 1.123–2.563)] events independently of age, serum and HDL cholesterol, cigarettes, BMI and SBP in the Gubbio study. However, there was no increased risk of CVD, CHD or STR in men with OWCET neither in the Gubbio 20-year follow-up nor in the IRA 50-year follow-up. These results were not modified significantly by the correction of the regression dilutions bias between the first and the subsequent SBP measurements. Thus, in primary care, OWCET should be actively evaluated in women as it can improve stratification of long-term CVD, CHD and STR risks.

## Introduction

Whereas an increase of 10 mmHg in systolic BP (SBP) can almost triple the relative risk for cardiovascular disease (CVD)^1–3^, the potential presence of the white coat effect (WCE)^2,4^ may interfere with these changes during office visits. We have previously described a proxy of WCE obtained during a family medicine visit^5^. It corresponds to the waxing and waning of BP and is defined as a ≥ 10 mmHg difference between the measurement made at the beginning and that made at the end of the visit. We named it ‘office white coat effect tail’ (OWCET) and showed that OWCET as a dichotomous variable, in the context of the large Gubbio residential cohort study with long-term follow-up, may interact with the exact measurement of BP and with the treatment of high BP, independent of several traditional risk factors including SBP^5^. By multivariate Cox model, a tendency towards increasing the risk by OWCET could also be seen for the incidence of stroke (STR) and for CVD and coronary heart disease (CHD) deaths but not all-cause deaths. However, in all instances sex was coded as a dichotomous variable and the risk was constantly higher in men^5^, so that whether OWCET could improve risk prediction in the population at large and in both sexes remained unknown.

In primary prevention, risk factors in women are treated less aggressively than in men^5–10^: Delpech *et al*. have previously shown that the SCORE cardiovascular risk was assessed less frequently in women than in men (OR = 0.63 [0.5–0.8]). A similar difference has been shown in secondary prevention. By the present study, we aimed specifically at investigating the impact of sex on OWCET in the incidence of long-term major fatal and non-fatal events (CVD, CHD and STR) in two Italian residential cohorts [Gubbio and the Italian Rural Areas of the Seven Countries Study (IRA)]. Moreover, we asked whether the regression dilution bias (RDB)^1^ might interfere, sex-wise, with OWCET and we therefore looked also at this to corroborate the results in women versus men.

## Methods

### Population and baseline examination

**Figure 1:**
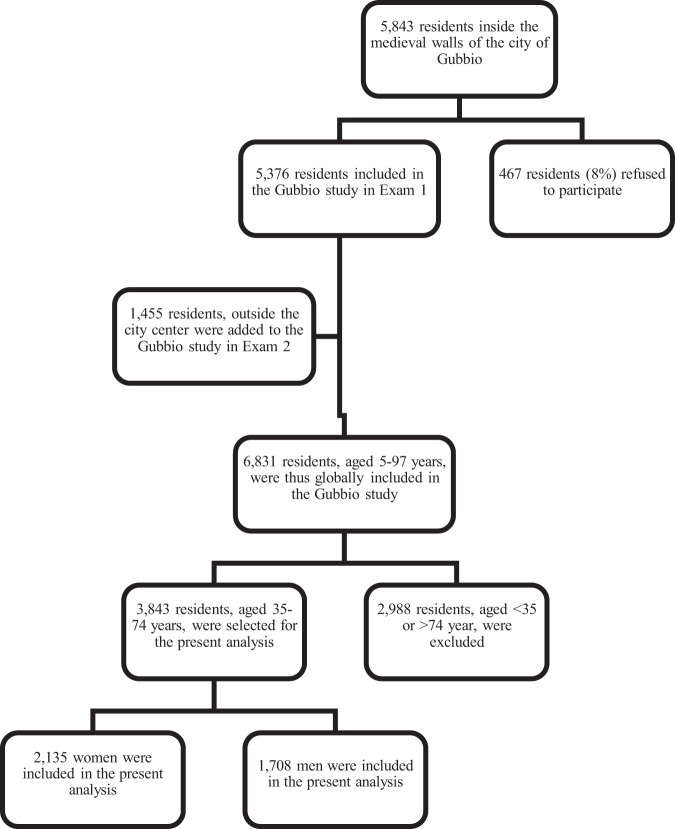
Flow charts of the Gubbio Study.

The methods and main results of the Gubbio residential cohort Study have been previously reported^11^. In brief, Gubbio is a hill town, in central Italy, at the lower reaches of the Apennine mountain chain. After approval by the regional ethical committee (see Declarations), an invitation to participate in the study was sent to the entire population aged 5 years and over resident inside the medieval walls (Fig. 1: 5843 individuals). Baseline examination (Exam 1) was performed between 1983 and 1986 after ethical approval by the Ethical Committee of the Local Health Authority of Alto Chiascio (Perugia, Italy) which was followed by the Ethical Committee of the Regional Authority of Umbria (reference #2850/16). A total of 5376 individuals participated in Exam 1. The first follow-up examination (Exam 2) was performed from 1989 to 1992. The invitation to participate in Exam 2 was sent to all individuals in the Exam 1 cohort. Of the 5376 individuals in the Exam 1 cohort (response rate 92%), 418 had died before Exam 2; 3727 individuals participated in Exam 2 (response rate 75%). For exam 2, an additional cohort of 1455 residents outside the city center either volunteered or were invited in order to complete the genealogies of their family. For the purpose of the present study, the Exam 1 cohort and the latter cohort were combined in the analyses.

A comprehensive baseline medical examination was performed including a battery of standard laboratory tests and measurements of special interest to the Gubbio Study in view of its focus on high BP^11^. Verbal consent was obtained from participants in compliance with the Helsinki Declaration. A questionnaire on lifestyle and health problems was administered, and a number of anthropometric, biochemical, biophysical and medical measurements were taken. The data presented here are limited to individuals aged 35 through 74 years in cohort 1 and in cohort 2. The following variables measured at the entry examination were used for the present analysis: age, sex, SBP and diastolic BP (DBP) measured according to a standardized procedure (see below), total serum cholesterol, high-density lipoprotein (HDL), smoking status, body mass index (BMI). Biochemical measurements were assayed by enzymatic methods: they were performed partly under the external control of the WHO Lipid Reference Center of Prague. In separate analyses (detailed results not reported) we used also fasting blood glucose (FBG).

**Figure 2:**
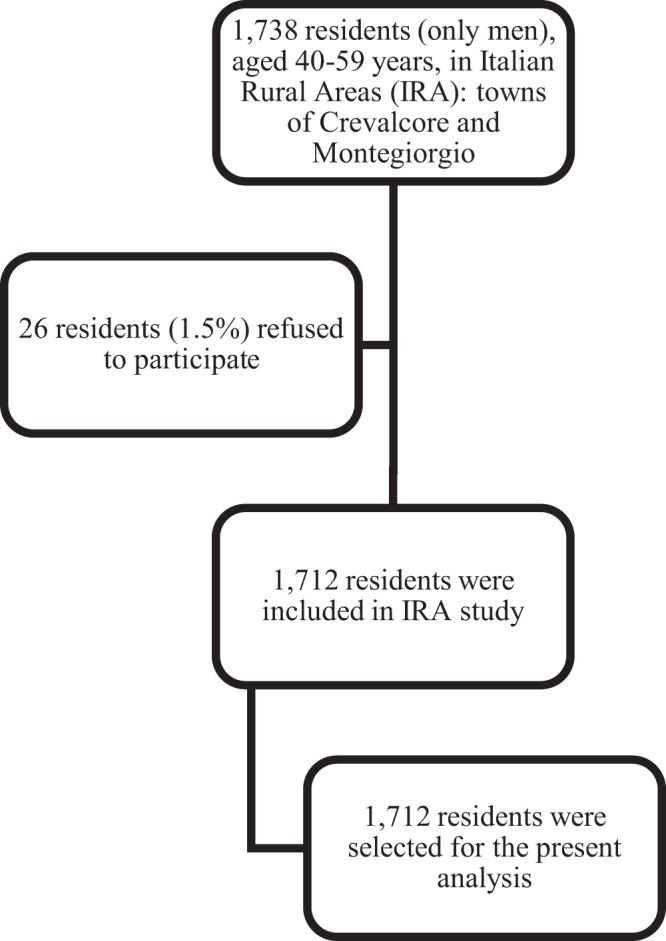
Flow chart of the Italian Rural Areas Study.

Individuals from the IRA study were only men of two villages of Northern (Crevalcore) and Central Italy (Montegiorgio), the latter not far from Gubbio. The analysis refers to baseline data of entry field examination held in 1960 (Fig. 2), when the cohorts were aged 40–59 years, with a participation rate of 98.5%, and a follow-up for mortality of 50 years^12^. The 45 baseline variables included age at baseline, family and social indicators, skeletal and anthropometric measurements, biophysical and biochemical measurements, clinical observations and diagnoses of selected major diseases. The following information and measurements taken at the entry examination were used for the present analysis: age in years, approximated to the nearest birthday, sex, SBP according to a standardized procedure (see below), serum cholesterol in mmol/L measured on a casual blood sample, smoking status (the number of cigarettes smoked on average per day, recorded from a standard questionnaire: 0 cigarettes for ex and never smokers), BMI expressed in kg/m^2^, derived from height and weight measured according to the technique described in the WHO Cardiovascular Survey Methods manual^13^. The Abel-Kendall method modified by Anderson and Keys was used for cholesterol measurements^14^. Baseline data were collected in the early 1960s before the era of the Helsinki Declaration. Later, oral informed consent was obtained in view of collecting follow-up data.

### Blood pressure measurements

In the Gubbio cohort, BP was measured by a physician with the participant in a sitting position, on the right arm positioned at heart level, using a mercury sphygmomanometer with appropriate-sized cuffs. Three measurements were taken following the procedure described in the 1975 WHO Manual^15^. The first measurement was taken at baseline after 5 min rest, then the remaining two measurements were taken approximately 1 minute each apart while the physician remained in the office. Measurements were made by four physicians who had undergone a specific training in BP measurement at the Istituto Superiore di Sanità (National Institute of Health) in Rome and at the London School of Hygiene and Tropical Medicine. The average of the second and third BP measurement was considered the actual BP, and it was used for classifying hypertensive and non-hypertensive subjects at baseline. The first Korotkoff phase was used to indicate SBP and the fifth phase was recorded as DBP; the same procedure was used in all screenings; observers were trained and tested for standardized procedures.

In the IRA cohort, two measurements were taken 1 minute apart and both were recorded at baseline. Two BP measurements were taken in supine position, at the end of a physical examination, using mercury sphygmomanometers and following the procedure described in the 1968 WHO Manual^13^. The average of the two measurements was considered the actual BP, and it was used for classifying hypertensive and non-hypertensive subjects at baseline.

### Office white-coat effect tail (OWCET)

The three and two BP measurements taken respectively at the initial visit in the Gubbio and in the IRA studies were used to define OWCET as a decrease in SBP between the first and the third measurement ≥10 mmHg in the Gubbio study or between the first and the second SBP measurement ≥10 mmHg in the IRA study. Individuals with smaller SBP changes were considered as OWCET negative. OWCET positive individuals were considered proxy^5^ to those having the classical WCE described by Mancia *et al*. using an invasive method, from the pre-medical appearance to the disappearance of the Doctor, several minutes later^16^. However, in order not to overestimate the phenomenon, we selected a higher difference as compared to the observation of 8 mmHg between the peack intraarterial phase and its tail as graphically reported by Mancia *et al*.^16^. Moreover, a 10 mmHg cut-off is an easy value to remember and corresponds to the quantity considered as a long-term CVD risk factor as related to SBP^1^.

### Follow-up and outcomes definition

In the Gubbio study, the follow-up was conducted during approximately 20 years (median 185 months), lasting from 13.5 to 19.5 years after the entry examination depending on the cohorts involved (either Exam 1 or 2). In the IRA study, the follow-up was obtained at quinquennial re-examinations (until year 40 of follow-up, except for year 15) and performing systematic searches of possible new events during 50 years.

In both studies, CV events were identified using data collected in subsequent field examinations and for those who did not attend, review of hospital records, interview with subjects, family physicians and telephone interviews. Home visits and postal questionnaires were performed only in the IRA study. Subjects were classified as lost to follow-up after systematic telephone search, home visits, and review of local municipality registers assigning a date when presumably last seen alive. Three end-points were considered for this analysis (5): (1) incidence of hard criteria of CVD, including CHD, non-fatal and fatal STR, non-fatal and fatal peripheral artery diseases (PAD) and non-fatal and fatal heart diseases of uncertain etiology (HDUE) including cases of heart failure, severe arrhythmias and transmission blocks in the absence of a clear CHD with typical characteristics. Cases characterized only by angina pectoris, intermittent claudication and transient ischemic attacks were not classified as hard events; (2) incidence of CHD, including non-fatal and fatal myocardial infarction and sudden coronary death (when other causes could be reasonably excluded); (3) non-fatal and fatal STR for which it was not possible to dissociate ischemic versus hemorrhagic strokes. Other rare cases of heart disease of defined etiology were not considered for analysis. The same criteria described above were used to identify prevalent cases of CVD recorded at entry examination that were excluded from analysis, together with other less common heart diseases, when appropriate.

In all cases, only the first event was used for the analysis. Major cardiovascular events were coded following the 9th Revision of the WHO-International Classification of Diseases^17^ for the Gubbio study and 8th Revision of the WHO-International Classification of Diseases for the IRA study^18^. Details on the diagnostic procedures and criteria are reported elsewhere for Gubbio^19,20^ or IRA studies^21,22^.

### Statistical analysis

Briefly^5^ data were expressed as means ± SD or percentage. Comparisons between groups were done by Student’s t-test or chi^2^-test, where appropriate. Survival curves were estimated using the Kaplan-Meier product-limit method and were compared by the Mantel log-rank test. The effect of selected covariates on survival was evaluated by Cox regression models (Efron ties method) using NCSS version 9 (Hintze J, Kaysville, Utah, USA: www.ncss.com). Models with Breslow’s approximations were also run for comparisons. Adjusted hazard ratios [HR ± 95% confidence intervals, (CI)] were calculated sex-wise by considering 7 covariates (age, BMI, total cholesterol, HDL, cigarettes smoked per day, SBP, OWCET) in the Gubbio study and 6 covariates (age, BMI, total cholesterol, cigarettes smoked per day, SBP, OWCET) in the IRA study. In a side analysis we studied the effect of correcting the RDB on the predictive role of SBP, comparing the first with one of the subsequent measurements, to explore the possibility that the phenomenon under study could be accordingly influenced. We used a variant of the MacMahon *et al*. method^1^ where the *dilution factor* was computed by dividing the difference in mean BP between the lowest and the highest quintile of the distribution based on the first BP measurement, by the same difference based on blood pressure levels at successive measurements. The correction of the regression dilution was made by multiplying the *dilution factor* by the original multivariate coefficient of BP and subsequently re-calculating the risk.

### Ethics approval and consent to participate

The Authors declare that all methods were carried out in accordance with relevant guidelines and regulations. The study of Gubbio adheres to the Declaration of Helsinki. Ethical approval was given by the Ethical Committee of the Local Health Authority of Alto Chiascio (Perugia, Italy) which was followed by the Ethical Committee of the Regional Authority of Umbria (reference #2850/16). Informed written consent was given by and filed from all participants. The study was chaired by a steering committee until early 2016 when the Centro Studi Epidemiologici di Gubbio (CeSEG) came into effect to chair it, under the auspices of the Italian Authority for Privacy. For the IRA study, baseline data were collected in the early 1960s before the era of the Helsinki Declaration. Later, oral informed consent was obtained in view of collecting follow-up data.

## Results

In the Gubbio Study there were highly statistically significant (p < 0.00001) differences, both in women and men, in the distribution of the SBP and of the difference between the third and the first SBP measurement used to define OWCET between subjects categorized as negative or positive OWCET. These were, respectively for SBP [women (N = 1582 versus 419): 135 ± 23 versus 142 ± 23 mmHg; men (N = 1290 versus 272): 133 ± 21 versus 139 ± 20 mmHg] and SBP3-SBP1 [women (N = 1582 versus 419): −1.6 ± 5.1 versus −13.8 ± 4.5 mmHg; men (N = 1290 versus 272): −1.5 ± 4.7 versus −13.4 ± 3.9 mmHg]. Moreover, women with positive OWCET were older than those with negative OWCET (57 ± 10 versus 54 ± 11 years, p < 0.00001).

**Figure 3:**
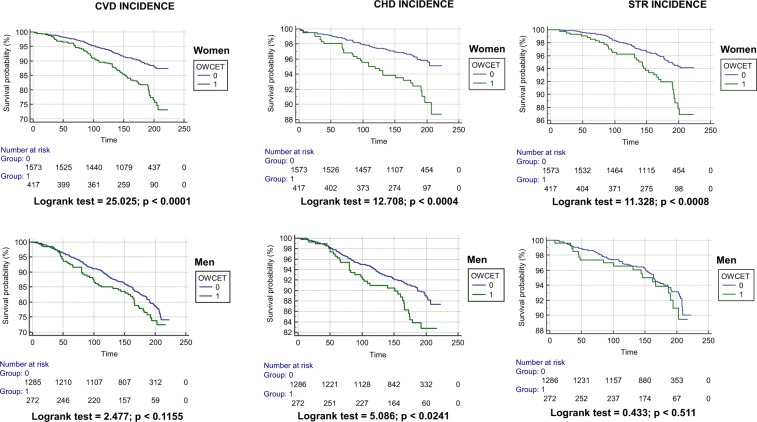
Kaplan-Meier graphs of the 20-year incidence of crude CVD, CHD and STR among women (*upper panels*) and men (*lower panels*) from the Gubbio residential cohort according to the absence (Groups 0) or presence (Groups 1) of office white coat effect tail (OWCET). While the univariate difference between OWCET groups was statistically different for all end-points in women, in men it was so only for CHD incidence.

Figure 3 shows the Kaplan-Meier curves of median 185-month follow-up in the Gubbio Study, separately for women (upper panel) and men (lower panel), respectively for the incidence of CVD, CHD and STR. OWCET negative and positive women had highly significant univariate differences (p < 0.001) for all outcomes with significantly higher risks among the latter group whereas in men there was only a significant (p < 0.05) univariate difference for CHD incidence.

After adjustment for age, SBP, total and HDL cholesterol, cigarettes and BMI, positive OWCET remained significantly associated with a higher risk of CVD, CHD, and STR in women, but not in men (Table 1). We separately performed different analyses (data not shown) either including prevalent cases or adding FBG as a covariate, both with and without prevalent cases but no deviations were seen from the solutions illustrated in Table 1. In all these different analyses, the contributory and statistically significant role of OWCET was confirmed in women but not in men.

**Table 1:** Hazard ratios (HR) and 95% confidence intervals (CI) of forced Cox model solutions (Efron’s ties method, although Breslow’s approximation gave similar results), computed on units of covariates, to predict incident events (fatal and non-fatal) in both sexes of the Gubbio population Study during 185 months of median follow-up.

	Means		Women (♀)^ª^						Men (♂)^ª^					
			CVD		CHD		STR		CVD		CHD		STR	
			HR	95% CI	HR	95% CI	HR	95% CI	HR	95% CI	HR	95% CI	HR	95% CI
**Covariates**														
	♀	♂												
Age (years)	54.14	52.99	**1.111**	1.092–1.131	**1.082**	1.053–1.111	**1.121**	1.090–1.153	**1.065**	1.051–1.079	**1.027**	1.009–1.045	**1.120**	1.090–1.152
SBP (mmHg)	136.39	133.87	**1.008**	1.003–1.015	1.007	0.998–1.017	**1.011**	1.003–1.020	**1.017**	1.011–1.022	**1.019**	1.011–1.027	**1.010**	1.001–1.020
TC (mg/dL)	225.47	223.75	1.001	0.997–1.004	**1.006**	1.001–1.011	1.001	0.995–1.005	1.002	0.999–1.005	**1.006**	1.003–1.009	0.999	0.995–1.005
HDL (mg/dL)	51.33	44.03	**0.979**	0.968–0.991	**0.967**	0.949–0.986	0.985	0.968–1.003	0.993	0.982–1.004	0.988	0.973–1.004	1.015	0.998–1.033
Cigarettes (N/day)	2.47	7.74	1.020	0.990–1.051	**1.042**	1.004–1.081	1.004	0.954–1.057	**1.023**	1.012–1.034	**1.027**	1.013–1.040	1.012	0.988–1.038
BMI (units)	27.43	27.25	0.995	0.966–1.004	0.990	0.944–1.039	0.973	0.931–1.018	**1.043**	1.010–1.077	1.041	0.997–1.087	**1.066**	1.006–1.129
OWCET (no: 0; yes: 1)	0.21	0.17	**1.591**	1.204–2.103	**1.614**	1.037–2.512	**1.696**	1.123–2.563	0.982	0.733–1.317	1.189	0.810–1.747	0.902	0.530–1.535
**Parameters**														
Missing x’s			68		68		68		24		24		24	
Processed			1934		1934		1934		1539		1539		1539	
Failed			223		89		100		287		152		91	
Censored			1711		1845		1834		1252		1387		1448	
Log likelihood			−1481.40		−605.11		−653.01		−1898.44		−1031.03		−566.03	

SBP: systolic blood pressure (the average between the second and third measurement); TC: total cholesterol; HDL: high-density lipoprotein cholesterol; BMI: body mass index; OWCET: office white coat-effect tail coded for >10 mmHg lower difference between the third and the first index SBP measurement; CVD: cardiovascular disease incidence by hard criteria; CHD: coronary heart disease incidence by hard criteria; STR: stroke incidence. When 95% CI do not cross 1, p < 0.05 (in bold). ªSolutions were done without eliminating prevalent codes and also by eliminating them but having glucose blood levels as a further predictor (mostly not significant due to an increased number of missing X’s up to 327 in women and 219 in men). However, no significant deviations were seen of the sex-related results of OWCET that were significant in women (HR from 2.052 to 3.410) and always not significant in men.

**Figure 4:**
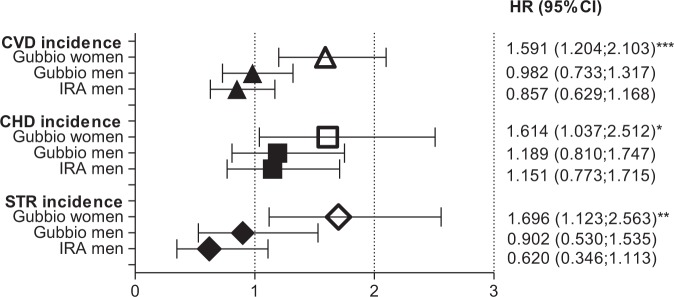
Hazard rates [(HR and 95% confidence intervals (CI)] for the incidence of CVD, CHD and STR in the Gubbio population Study (over 20 years) and in the Italian Rural Areas of the Seven Countries Study (over 50 years), adjusted for age, SBP, total and HDL cholesterol (only total cholesterol in IRA), cigarettes and BMI. Sexes are separated but only men are present in IRA. Note that HR is significant (p < 0.001 to 0.05: asterisks) only in women for all end-points.

In the IRA study, that included men only, we found that positive OWCET did not increase the risk of CVD, CHD, and STR (Fig. 4) yet was slightly different from the one observed in the Gubbio Study and seen in a smaller proportion (5.22%) of the male population, it was always not a significant contributor (HR for CVD in 50 years: 0.857 and 95% CI 0.629–1.168, p = 0.330; CHD in 50 years: 1.151 and 95% CI 0.773–1.715, p = 0.489; STR in 50 years 0.620 and 95% CI 0.346–1.113, p = 0.109) and in ranges superimposable to the ones seen in men from the Gubbio Study. Traditional risk factors in IRA, like the ones investigated in the Gubbio study (but except HDL cholesterol that was not assessed in the IRA cohort), were expectedly predictive of all outcomes (Table 2).

**Table 2:** Hazard ratios (HR) and 95% confidence intervals (CI) of forced Cox model solutions (Efron’s ties method, although Breslow’s approximation gave similar results), computed on units of covariates, to predict incident events (fatal and non-fatal) in both genders of the IRA Study during 50-year of follow-up.

	Means	Men (♂)ª					
		CVD		CHD		STR	
		HR	95% CI	HR	95% CI	HR	95% CI
**Covariates**							
	♂						
Age (years)	49.10	**1.090**	1.074–1.106	**1.050**	1.029–1.072	**1.081**	1.055–1.107
SBP (mmHg)	144.13	**1.014**	1.010–1.018	**1.009**	1.004–1.014	**1.013**	1.007–1.019
TC (mg/dL)^b^	201.61	**1.003**	1.002–1.005	**1.005**	1.003–1.007	**1.002**	0.995–1.005
**Cigarettes**							
(N/day)	8.74	**1.011**	1.004–1.018	**1.013**	1.003–1.022	0.998	0.986–1.011
BMI (units)	25.20	0.987	0.968–1.008	0.990	0.944–1.039	0.978	0.945–1.013
**OWCET**							
(no: 0; yes: 1)	0.05	0.857	0.629–1.168	1.151	0.773–1.715	0.620	0.346–1.113
**Parameters**							
Missing x’s		102		102		102	
Processed		1610		1610		1610	
Failed		903		447		323	
Censored		707		1163		1286	
Log likelihood		−5744.55		−2973.25		−2104.16	

SBP: systolic blood pressure (the average between the first and the second measurement); TC: total cholesterol; BMI: body mass index; OWCET: office white coat-effect tail coded for >10 mmHg lower difference between the second and the first index SBP measurement; CVD: cardiovascular disease incidence by hard criteria; CHD: coronary heart disease incidence by hard criteria; STR: stroke incidence. When 95% CI do not cross 1, p < 0.05 (in bold). ªSolutions were done without eliminating prevalent codes. However, no significant deviations were seen as compared to those reported here. ^b^Note that HDL cholesterol was not measured in the IRA Study, differently from the Gubbio Study.

Results on the analysis of RDB are presented synthetically in Table 3 where it appears that the correction of the possible dilution bias does not change the predictive power of the first SBP measurement in the presence of OWCET. Instead, OWCET contribution (detailed results not shown) was always significant and evident in women but not in men also when RDB was taken into account. Specifically, when comparing the solutions with the first SBP measurement and the average SBP in women, the t of the coefficients’ difference was −0.0471 (NS) whereas the log-likelihood of the original model with the average SBP (and OWCET) in the Gubbio women was −1481.3965 compared to −1481.7650 of the model containing the first SBP measurement and OWCET.

**Table 3:** Comparison of original models with a model where the multivariate SBP coefficient was adjusted for the regression dilution bias (RDB) bound to the subsequent measurements.

	Gubbio Women	Gubbio Men	IRA Men
**Original model including with average SBP and OWCET**			
Relative risk	41.0	10.4	1.18
ROC	0.815	0.636	0.542
**Model with first SBP and OWCET**			
Relative risk	41.0	12.5	1.18
ROC	0.815	0.732	0.542
**Model including first SBP (using a multivariate coefficient adjusted for RDB) and OWCET**			
Relative risk	41.3	9.8	1.17
ROC	0.815	0.732	0.542
Increase of SBP coefficient by regression dilution factor	8.8%	10.0%	4.6%

Relative risk = ratio between quintile 5 and quintile 1 of the estimated risk.

ROC: receiver operating characteristic curve.

## Discussion

### Main findings

The main finding of the study was that in apparently healthy women of the Gubbio residential cohort OWCET predicted CVD, CHD and STR incidences during approximately 20-year follow-up, independent of standard risk factors whereas this was not the case in men (Table 1). Among men, this negative result was externally validated in the IRA study at 50-year follow-up (Fig. 4). When the dichotomous covariate was forced into a multivariate Cox model among Gubbio women, it increased the hazard of incidence of CVD, CHD and STR by 59 to 70% whereas in men no significant change occurred. The correction of RDB of first versus second measurements did not modify significantly these results. Incidentally, women with positive OWCET were significantly older than those with negative OWCET.

When assessing the incidence of CVD, CHD and STR in a previous investigation on this material^5^, it was observed that male sex always contributed significantly (HR from 1.459 to 2.116) whereas OWCET increased by 25 to 35% the risk of incidence of CVD and CHD but not of STR incidence. The interaction term sex*OWCET was significant for CVD only (HR 0.626 and 95% CI 0.418–0.937, p = 0.0229) and borderline for STR incidence (HR 0.528 and 95% CI 0.270–1.039, p = 0.0625) whereas again sex was always a contributor (HR from 1.721 to 2.064) as was OWCET (HR from to 1.579 to 1.654) calculated separately. These preliminary results indicated the need of exploring sex-wise relations of OWCET, what was done specifically in the present investigation.

### Sex-related relationships

Manios *et al*. showed by multiple linear regression models, among 2004 Greek subjects who had undergone office BP measurements and 24-hour ambulatory BP monitoring (ABPM) on the same day, that female sex (beta = 0.166; p < 0.001), age (beta = 0.039, p = 0.020), office SBP (beta = 0.727; p < 0.001), daytime SBP variability (beta = 0.128; p < 0.001) and smoking (beta = 0.031, p = 0.048) are independent determinants of systolic WCE^23^. Segre *et al*. had previously demonstrated a significant correlation (p < 0.05) between WCE and sex among 670 Brazilian patients^24^. Finally, Streitel *et al*. investigated more recently whether sex was a unique predictor of the WCE in a population of normotensive and high BP patients^25^. The association between sex and the systolic WCE was small in 252 individuals from the US and it was likely accounted for by other variables including age, BMI, state of anxiety, and household income to conclude that sex may be of limited use in helping identifying patients who may be more likely to have WCE. Our study on a true residential population showed that female sex is a significant factor to separate the impact of OWCET in CVD, CHD and STR outcomes multivariately. On the other hand, women with positive OWCET in the present investigation were older by around 3 years than those without and the age difference might have an impact since mechanistically, positive OWCET may be ascribed to arterial stiffness as in the case of WCE.

It is unclear why OWCET is an independent predictor of long-term outcomes among women and not among men. We have no actual means of dissecting this from our study, apart the age difference between women with or without OWCET, and therefore further investigations are warranted: they deserve to concentrate on pathophysiological determinants of BP levels including arterial stiffness and to responses to stimuli such as efforts, emotions and activation of sympathetic and parasympathetic systems either directly or indirectly via psychological interferences.

### The impact of WCE/OWCET on CVD risks

There were very few previous reports to assess the impact of WCE on CVD risks. Verdecchia *et al*. in a relatively short-term study performed in the same Italian region as that of the present investigation showed that the rate of fatal and non-fatal CVD events was not increased among hypertensive patients with WCE but sex-wise relationships were not explored^26^. Franklin *et al*.^27^ showed in an international case-control study of 1306 subjects that the size of WCE is independent of CVD risk. There are notable differences with the present related to the predictive models used (linear versus exponential), the different types of study (case-control versus longitudinal ones in residential cohorts), the follow-up duration [in general much shorter in all former studies^26,27^] and especially in the peculiarity whereby risk factors were considered, not assessing them as continuous covariates.

### Regression dilution bias

RDB (or regression to the mean) is a generally recognized statistical phenomenon that occurs when repeated measurements are made on the same subject or unit of observation^1^. It happens because values are observed with random error. A random error is a non-systematic variation in the observed values around a true mean (e.g. random measurement error, or random fluctuations in a subject). Systematic error, where the observed values are consistently biased, is not the cause of RDB. It is rare to observe data without random error, which makes RDB an ubiquitous phenomenon. There are several methods to compute RDB and we selected one among others. The essential of the result was (Table 3) that RDB did not interfere with the relevance of OWCET among women and we should accordingly conclude that no significant contribution might exist. On the other hand, the time difference between measurements was much shorter than that commonly used in this kind of approach.

### Limitations

The Gubbio and IRA studies did not perform ABPM neither was BP measured at home. For the Gubbio study, standard risk factors were measured in the context of an epidemiological investigation in the 80’s which had, among its goals, that of assessing the impact of community control of BP and the relationships between mortality and changes in CVD risk factor levels although the use of questionnaires may well be considered a limitation^28^. On the other hand, the IRA study was made to identify CVD risk factors at the beginning of the sixties and ABPM did not exist at the time. Moreover, the analysis and interpretation of the IRA data might have slightly different meaning compared to those of the Gubbio study. In fact, in the IRA study only two BP measurements were recorded (versus three in the Gubbio study) but at the beginning of the 1960’s recording two measurements was already an exception in most studies. Clearly, the different methods used and the fact that only men were included in the IRA Study should be considered among the limitations of this study. Other differences between the two studies were the measurement of BP in supine position in the IRA and in sitting position in the Gubbio cohort and the lack of HDL measurement in the IRA cohort (Table 2). Finally, both investigations were carried out in an historical period where primary care did not foresee the potential presence of a specific care manager who may indeed provide an advantage^29^ and recognize individuals deserving special attention also on the basis of individualized and sex-specific OWCET.

### Conclusions

The idea of assessing the potential contribution of OWCET, a proposed proxy of WCE^5^ comes from the methods whereby the BP measurements were obtained in the Gubbio residential cohort, namely spaced measurements with time intervals between the first and the third one. We have shown here an independent predictive role of OWCET on approximately 20-year CVD, CHD and STR incidences in women but not in men, without any statistically significant contribution related to the correction of the RDB. The negative result obtained among Gubbio’s men was externally validated in a second Italian residential cohort. It is therefore recommended that clinical visits of women in primary care have these measures taken which might help for prognostication. Older women should be paid more attention in this regard. However, these results should be repeated elsewhere to confirm their sex-wise relevance. It should also be important to assess arterial stiffness to see whether this is related to OWCET age-dependently.

## Data Availability

Data might be requested to CeSEG provided obtaining the authorization by the Italian Authority for Privacy.
